# How does the media contribute to the rise of hate crimes against foreign domestic helpers in Hong Kong? An unfair problem frame and agenda setting

**DOI:** 10.3389/fsoc.2024.1374329

**Published:** 2024-05-30

**Authors:** Zhuoli Chen

**Affiliations:** Department of Sociology, The University of Hong Kong, Hong Kong, Hong Kong SAR, China

**Keywords:** hate crimes, agenda setting, foreign domestic helpers, Hong Kong, problem frame

## Abstract

Hate crimes are widespread in Hong Kong society. Foreign domestic helpers working in Hong Kong also experience unfair agenda-setting by the media due to their dual economic and social disadvantages, and the media tries to portray them in a hostile social role. At the same time, the media creates negative social images of minority groups through news coverage, which leads to an increase in social hate crimes against them. This study used WiseSearch, a Chinese newspaper collection and analysis platform, to explore how Hong Kong news media use news themes and content to create a negative image of Hong Kong foreign domestic helpers in order to understand the media origins of hate crimes against Hong Kong foreign domestic helpers. Ultimately, the study found that local news media in Hong Kong are more inclined to cover the legal disputes of foreign domestic helpers in the agenda-setting process. In addition, they are more likely to associate foreign domestic helpers with “fear” rather than “rest assured.” The study also found that because of the news value orientation, Hong Kong media tended to treat foreign domestic helpers as outsiders and less sympathetically when writing news stories.

## Introduction

### Hate crimes

Hate crimes are generally defined as unlawful, violent, destructive, and threatening behaviors against a presumed social group of the victim due to the offender’s prejudice against them (Green et al., 2001). A study states that the terms hate crime and bias crime were coined in the United States in the 1980s. The underlying reason for their creation was that journalists and policy advocates were trying to come up with new terms to describe violence against Judeans, Black people, and Gay men (Green et al., 2001). However, at the same time, hate crimes are not only acts of violence but also offenses such as vandalism, harassment, and trespassing. We could define hate crime on different levels; firstly, in terms of the group of victims, the broadest definition currently available suggests that hate crime includes words or actions intended to harm or intimidate an individual; in short, the group of people who are targeted by hate speech are also the victims of hate crime (Green et al., 2001). Secondly, in terms of the form of the offense, the scope of hate crime is vast, starting from the minor form of hate speech, and any unlawful act directed at a specific group of people may fall within the scope of hate crime (Green et al., 2001).

Having explored the comprehensive definitions and varied forms of hate crimes, we now turn our attention to a specific and persistent issue: hate crimes against foreign domestic helpers (FDHs) in Hong Kong. Hate crimes against FDHs in Hong Kong are a long-standing problem and have even evolved into social exclusion. A survey of Filipino FDHs in Hong Kong showed that 20.5% of the respondents had suffered physical abuse, and 34.4% had suffered verbal abuse in the past 12 months (Cheung et al., 2019). At the same time, FDHs were more likely to suffer from depression, higher than the general population of Hong Kong. Not only that, but due to unfair policies and structures, Hong Kong FDHs are afraid of losing their jobs, so in the face of abuse and violence, 16.7% of victims do not report the crime (Cheung et al., 2019). Among the total number of cases reported, only 19.4% of the people report the crime to a formal agency or the police (Cheung et al., 2019). The staggering statistics from this survey of Filipino FDHs in Hong Kong shed light on their harsh reality. Furthermore, the findings indicate that a specific social mechanism is contributing to the challenges faced by FDHs. The objective of this article is to examine the nature of this social mechanism and its operational characteristics.

Moreover, the impact of media portrayal becomes evident in studies that reveal how traditional media contributes to the dehumanization and social exclusion of FDHs. Some studies have found that when reporting on legal cases involving FDHs, the Hong Kong media tend to portray them as foreigners with defective characters, leading to dehumanizing treatment of FDHs and thereby becoming morally ostracized by society (Ladegaard, 2013). The above studies have proven the social environment where FDHs in Hong Kong could be friendlier. At the same time, relevant reports from traditional media are exacerbating this phenomenon. Moreover, a moral panic about foreigners may prompt the public to demand unprecedented new legislation and strengthen law enforcement practices (Zatz and Smith, 2012). However, strengthening law enforcement against foreigners may lead to an increase in their crime rate, which negatively confirms the public’s attitude toward them. This, in turn, may cause their social image to fall into a vicious cycle shaped by problem frame (Zatz and Smith, 2012).

As we delve into the complex landscape of hate crimes, it becomes apparent that social factors such as race, class, and gender play a pivotal role in motivating these crimes. This is especially pronounced in cases like hate crimes against FDHs, where societal biases intersect with discriminatory practices. Furthermore, the media emerges as a powerful force in shaping the motivations behind hate crimes, influencing public perceptions, and exacerbating existing prejudices based on race, class, and gender.

In point of fact, the existence of unfair mechanisms, cultural contexts, and biased hate crimes all serve to illustrate a tragic narrative concerning FDHs.

Based on the above research, we restored a group portrait of FDHs like this: Those FDHs who relocated from their place of origin encountered both cultural and economic challenges in Hong Kong. Concurrently, the media has constructed a problem frame around issues pertaining to foreign domestic workers, which is based on exclusivity. As the problem frame gradually becomes agenda-setting, FDHs eventually encounter “social exclusion.” As FDHs are gradually regarded as “the others” by Hong Kong society, they further lose their limited social recognition and job opportunities. Such a situation may result in an increase in the crime rate among foreign domestic workers, thereby reinforcing the negative stereotypes associated with the problem frame.

In conclusion, the fluid nature of hate crime definitions and the stark realities faced by specific groups, such as FDHs, highlight the urgent need to examine the media’s role in perpetuating these crimes. The following discussion delves into how media contributes to the complex motivations and manifestations of hate crimes.

### Media bias and hate crimes

The impact of media bias on hate crimes is a recurrent theme in various studies across different geographical locations and contexts. For instance, a study conducted in the Netherlands by Jacobs and Van Spanje (2021) emphasized how biased media portrayal of minority groups, specifically news related to terrorism and immigration, contributes to an escalation in non-violent hate crimes, such as hate speech. This finding aligns seamlessly with the observations made in a United States-based investigative study covering hate crimes against Asian Americans from 2010 to 2022 (Powers et al., 2023). The study linked the surge in hate crimes against Asian Americans to misinformation about COVID-19 propagated by public officials and the media, underscoring the influential role of media narratives in shaping societal responses.

Moreover, the resonance between studies is not confined to specific cultural regions. In the United States, Robinson’s (2000) research highlighted how media coverage of crime targeting ethnic groups and perceived injustices in the criminal justice system fosters misperceptions and reinforces crime-related stereotypes for certain races. Simultaneously, a study in Austria by Schäfer and Schadauer (2018) indicated a correlation between the increase in racist incidents and a rise in online hate speech directed at refugees and immigrants, showcasing the global reach of media impact on hate-related phenomena. Expanding our perspective to Singapore, Lee and Thien’s (2015) study uncovered a noteworthy relationship between media reporting on crimes of a specific race and the severity of criminal penalties imposed on that race. This cross-cultural pattern emphasizes the pervasive influence of media narratives on societal perceptions and judicial outcomes. Furthermore, those articles provide an academic explanation for the social exclusion faced by FDHs in Hong Kong: Based on the Hong Kong media’s biased portrayal of FDHs, hate crimes and unfair judicial treatment against FDHs are increasing. These circumstances have resulted in serious consequences, ultimately leading to the social exclusion of FDHs and their categorization as “The Others.”

Simultaneously, a significant amount of research indicates that stereotypes regarding a specific ethnic group increase the probability of that group being targeted in hate crimes (Niang et al., 2003; Herek, 2009; Sanders, 2016; Bruns et al., 2020; Xu et al., 2021). Furthermore, studies conducted in Hong Kong have revealed that public culture and the perpetuation of stereotypes serve to legitimize the domestic abuse experienced by FDHs (Ladegaard, 2013). Those prejudices and stereotypes against FDHs in Hong Kong increase their vulnerability to hate crimes.

Parallelly, the Agenda Setting theory, as introduced by McCombs and Shaw (1972), sheds light on how media, through repeated reporting, binds negative issues to minority groups, molding public attitudes and fostering prejudices. This theory becomes particularly pertinent when examining the media’s biased agenda-setting in Hong Kong concerning reports related to FDHs. The potential repercussions include a surge in local hate crimes against FDHs, mainly manifesting in an uptick in hate speeches. In essence, these studies collectively underscore the significant influence media reporting holds in shaping public perceptions, demonstrating a clear correlation between biased media portrayal and the escalation of hate crimes against marginalized communities.

### Altheide’s research and problem frame

Furthermore, Altheide’s research on s and fear in media reporting provides a lens through which we can analyze how the Hong Kong media shapes perceptions of FDHs, potentially contributing to increased prejudice and hate crimes against this group.

Altheide’s research is based on the recognition of two basic social facts: (1) popular culture contains a relatively large number of messages and images related to fear, including crime and violence; and (2) the public perceives social life as very dangerous (Altheide, 1997). In other words what Altheide perceives as fear actually refers to members of society’s fear of crime and violence (Altheide, 1997). Similarly, Altheide’s definition of fear is also applicable to this study, i.e., the Hong Kong public’s fear of FDHs crime and violence, and such a definition also motivates us to focus on legal disputes and fear-related stories in the process of coding and defining news content (Altheide, 1997).

Crime reports almost reshape public perception, making the public think they are in danger. The plays a vital role in this process. Put, framing simplifies and reshapes vague and complex social issues, making them consistent with the experience and cognition of social members, making them easier to accept. However, problem frame s are also used by commercial media. In order to pursue higher ratings and views, news media entertainment methods are incorporated into regular social issue reports. This also gives the problematic framework the following characteristics:

1. It contains negative content;
2. the issue is relevant to the general public;
3. it is presented in a way that is easy to understand (simplifies the complex);
4. it has a problem that needs to be solved;
5. there are methods and mechanisms for solving the problems;
6. and the solutions to the problems are well known (e.g., policy approaches) (Altheide, 1997).

In order to comply with these content characteristics, news media pursue fear reporting, making it a common practice for commercial media to pursue ratings and attention. However, such fear reporting is also biased. For minority groups and groups that lack a voice, the news media also uses their status as a mainstream voice to construct society’s understanding of minority groups through. Fearful reports against minority groups distort society’s perception of their social role (Altheide, 1997).

Therefore, to apply Altheide’s framework to a concrete case, this study focuses on the media portrayal of FDHs in Hong Kong. Analyzing media reports, we aim to unravel how fear becomes intertwined with the image of FDHs through, contributing to societal biases and, consequently, an upsurge in hate crimes against this marginalized group. This study posits that the Hong Kong media’s use of fear, as identified by Altheide, plays a pivotal role in linking negative perceptions with FDHs, ultimately fostering an environment conducive to hate crimes against this vulnerable population.

### Research questions and coding criteria

#### Definition explanation

From the preceding discussion, we still need to clarify a few key definitions to set the stage for the research question. Firstly, framing refers to the process by which a set of created news content, shapes individual perceptions and behaviors (Moy et al., 2016). In contrast, Altheide’s (1997) problem frame refers to the process and manner in which the news media and other forms of communication present social problems in a way that emphasizes fear and danger. Agenda setting is what happens when increased media coverage of an issue leads to increased perceptions of the importance of the issue (Moy et al., 2016). Based on the above definitions, some researchers have pointed out that framing in media research focuses on the textual content and the frame itself, while agenda setting further considers the impact of the frame on the public (Maher, 2001). At the same time, although framing itself is a phase of agenda setting, Maher argues that the two should be studied separately due to the vast differences in the content of the two studies (Maher, 2001). In conclusion, this study sees problem frames as a way in which the media use the emphasis on fear and danger to shape social issues prior to agenda setting, but at the same time problem frames are also a phase of agenda setting.

#### Research questions

Based on the differing definitions above, our research question can be divided into two parts. Firstly, we will examine how the Hong Kong media set up the problem frame for FDHs by emphasizing fear. Secondly, we will explore how this problem frame influences and shapes public perception, ultimately leading to an unfair agenda setting.

However, it is important to acknowledge that the first part of the question can be analyzed through text to determine the extent to which the Hong Kong media associates fear with FDHs in the use of problem frames. As for the second part, which concerns the impact of the media phenomenon on society and the public, more in-depth research studies, such as in-depth interviews, are necessary to obtain information. Therefore, this brief initial study will focus solely on the first part to pave the way for further in-depth explanatory research. The second part will mainly highlight the connection between Problem Frame and agenda setting through a literature review, paving the way for further in-depth discussion.

#### Coding criteria for collected cultural products

Altheide’s study explored problem frames and fear through an analysis of 180 articles from the Los Angeles Times between 1985 and 1994, as well as 31 reports from ABC News broadcasts between 1990 and 1994. The term ‘fear’ (and other synonyms, such as afraid and threatened) was identified based on the date, year, title, key phrases, and location of its occurrence in these reports (Altheide, 1997). The study analyzed the meaning, usage, and significance of the term ‘fear’ through qualitative research conducted in groups (Altheide, 1997).

This study followed a similar strategy. Wisers owns WiseSearch, the leading big data database in the Chinese language. It includes all mainstream media (government-registered media) in Hong Kong and China and has a decade-long data retention. Advanced AI technology is applied for dynamic identification and data collation, making it our source of choice. WiseSearch was used to collect all news reports related to ‘domestic helper’ and ‘fear’ (including synonyms) in Hong Kong media, resulting in a total of 174 articles. Additionally, 4,032 articles related to ‘FDHs’ and ‘employees’ were analyzed in terms of headlines and article contents, as well as the location of the word ‘fear’, in order to group the articles accordingly and conduct a qualitative study.

## Methods

Based on the research purpose of this study, we aim to explore how mass media employs fear and problem frame to create biased perceptions of FDHs in pursuit of understanding how mass media contributes to biased perceptions of FDHs and the subsequent manifestation of hate crimes; this study employs two distinct research methods.

To investigate the issue comprehensively, we initially utilized WiseSearch, a big data platform aggregating news from Chinese media. The search involved using “domestic helper” and its synonyms as keywords, and we further examined the role of “fear” and “rest assured” within the identified articles. In the cultural context of Hong Kong, “domestic helper” specifically refers to foreign domestic helpers. For the initial search, we used “domestic helper” and synonyms as keywords in WiseSearch, covering titles and texts from all Hong Kong media sources within 1 year (10/15/2022–10/16/2023). The search yielded 2,233 news articles. Subsequently, we refined the search by examining the occurrences of “fear” and “rest assured,” resulting in 59 and 34 relevant articles, respectively. Following this, we conducted a one-month search for the term “employee” (from 2023/9/16 to 2023/10/15) under constant conditions, retrieving 2,932 reports as a comparison group. Finally, a three-year search (2020/10/16–2023/10/15) using the keywords “domestic helper” and “fear” analysis will reveal evolving patterns in the media’s framing of the topic through fear reporting and problem frame, with a total of 174 results.

In addition to the quantitative analysis, we employed a qualitative approach by selecting the top 10 most frequently covered stories related to “domestic helper “and “fear” from the 59 identified articles. To refine our focus, we engaged in content clustering, identifying similar articles based on content and title. This process involved counting them as a single article and eliminating those deemed irrelevant to the specific topic of “fear” about FDHs. We then extracted sentences containing the term “domestic helper” from the refined articles, identifying high-frequency words within these sentences. This vocabulary analysis aimed to unravel how the media employs question frames in shaping discussions related to FDHs. This study used ChatGPT 3.5 to check and touch up the article’s grammar.

## Results

### Step 1

Table 1 presents the classification outcomes of 2,233 articles from all Hong Kong media sources gathered over a year (2023/10/15–2022/10/16). The search involved the title and text, with “domestic helper” as the keyword and its synonyms considered. Articles with conspicuous topics were grouped accordingly, leaving 1,240 articles assigned to a conclusive topic category. It was discovered that approximately 50% of the articles surveyed related to legal disputes, with a total of 618 entries. Promotions were the second highest proportion, accounting for only about 8%. Notably, there was a significant difference between the highest and second-highest proportions.

**Table 1:** “Domestic helper.”

Subject name	Number of articles
Legal disputes	618
Promotion	96
Company notices	78
Hong Kong legal adjudication	78
Labour market and issues	71
Hong Kong civil affairs	66
Hong Kong health	64
New products	63
Labor/personnel	54
Corporate executives	52
All	1,240

Table 2 presents the classification outcomes of 2,792 articles about “employees “. Demonstrates the identical search scope, media authors, synonyms, and subject classification criteria utilized in the initial search using the keyword “employee.” The search encompassed all media sources in Hong Kong over 1 month (2023/10/15–2023/9/16) and yielded a total of 2,792 articles, which were subsequently subjected to topic classification. A total of 412 reports concerning legal disputes, which account for approximately 15% of the total proportion of reports, were found to be the most frequently reported topic. The second most commonly reported topic was articles featuring corporate executives as the subjects, accounting for around 14% of the total proportion.

**Table 2:** “Employee.”

Subject name	Number of articles
Legal disputes	412
Corporate executives	385
Senior management	385
Hong Kong Hang Seng Index	373
Labour/personnel	257
Labour market and issues	240
Board of directors	233
Labour laws and regulations	175
Executive interviews	167
Nasdaq	165
All	2,792

### Step 2

Table 3 displays a thematic classification of 57 articles about “fear” and “domestic helper.” Following identifying 2,233 articles through step 1, their titles and contents were examined for content relating to “fear,” encompassing considerations of synonymous terms, which resulted in their classification by subject. A thematic breakdown of 57 articles was derived, with 33 articles about legal disputes representing 58% of the total.

**Table 3:** “Fear” and “domestic helper.”

Subject name	Number of articles
Legal disputes	33
Hong Kong legal adjudication	6
Adverse reactions	4
Education in Hong Kong	3
Labour market and issues	3
Product efficacy	2
Hong Kong housing	2
Hong Kong civil affairs	2
Aviation or travel emergencies	1
Promotions	1
All	57

Meanwhile, Table 4 presents a thematic categorization of 34 articles relating to “rest assured” and “domestic helper.” The articles were identified through searches for content containing the phrase “rest assured” in the titles and contents of 2,233 articles, which were then classified by topic, taking into account synonyms. Eventually, 34 articles were obtained, of which eight were based on information about new products, accounting for around 24% of the total proportion.

**Table 4:** “Rest assured” and “domestic helper.”

Subject name	Number of articles
New product information	8
Brand activity	5
Hong Kong hygiene	4
Product reviews	3
Legal disputes	3
Promotions	3
Hong Kong civil affairs	3
Company announcement	2
Hong Kong education	2
Aviation or travel emergencies	1
All	34

Furthermore, Table 5 delves into high-frequency words identified in Hong Kong news articles discussing “domestic helper” and “fear.” We first identified the top 10 most frequently reported news stories, conducted content clustering, and refined the analysis to three stories most reported on the “fear” topic. Then, find the sentence containing “domestic helper” in the article and find the high-frequency words that appear in the sentence.

**Table 5:** High-frequency vocabulary.

	Article 1	Article 2	Article 3	Total
Crime (crime, confession)	10	2	0	12
Police (call the police, police)	4	0	3	7
Rape	7	0	0	7
Fear	0	1	4	5
Beat	0	0	3	3
Serious	3	0	0	3
Abuse	0	0	2	2

### Step 3

Having gained insights from thematic analyses in Step 2, we now move to Step 3, which involves a three-year (2020/10/15–2023/10/15) search using the keywords “domestic helper” and “fear.”

Table 6 reveals the outcomes of a comprehensive three-year search for articles discussing “domestic helper” and “fear,” using the same search conditions as the initial search. The results, obtained through content clustering, indicate a declining trend in reports concerning “domestic helper” and “fear” over the years.

**Table 6:** Media coverage of “domestic helper” and “fear.”

	2023/10/15–2022/10/16	2022/10/15–2021/10/16	2021/10/15–2020/10/16
Before content clustering	57	48	69
After content clustering	26	31	42

## Discussion

The research findings indicate that when searching with the term “domestic helper,” approximately half of the news relating to this term pertains to legal disputes, as per Tables 1, 2. Conversely, when searching with the term “employee,” only about 15% of the news concerns legal disputes. Given that FDHs are a type of employee, their news topic structure should align closely with that of other employees. However, there are stark differences between the two. News reports involving FDHs predominantly revolve around legal conflicts, which indicates that society holds a more unfavorable perception of FDHs compared to regular employees. At the same time, the content of legal disputes, as a manifestation of the loss of social order, is also very conducive to applying problem frames. When “domestic helper” became more linked to legal conflicts, the media pounced on the legal aspect of the “domestic helper” topic, resulting in legal disputes making up almost 50% of the total coverage. However, biased reports generated by these unjust agendas have influenced the public perception of FDHs, creating an impression that they are more prone to getting into trouble than the general population. Consequently, this has facilitated the development of negative emotions, fear, dissatisfaction, or apprehension toward them.

Having established the overarching findings, let us delve into a nuanced examination of specific keywords like “fear” and “rest assured,” shedding light on their impact on the portrayal of FDHs in media narratives. After comparing the data from Tables 3, 4, we conducted searches for the terms “fear” and “rest assured” separately out of the 2,233 collected articles. Our findings led to discoveries, specifically with regard to “fear,” where the percentage of articles on legal disputes rose to 58%, bringing the total number of articles to 57. When searching for “rest assured,” we found only 34 articles. Therefore, this means that under the topic of “domestic helper,” the possibility of news reports being related to the topic of “fear” is higher than that of “rest assured,” About 68%. Furthermore, the majority of the articles obtained by searching for “rest assured” are about new product information (8 articles) and brand activities (5 articles), indicating a weak correlation with “domestic helper.” Reports related to FDHs are not neutral. Instead, they tend to create unfair problem frames to associate FDHs with fear, creating content conditions for agenda-setting.

On the one hand, words such as fear and horror shape the characters in the news reports and create the image of the victim, making the crime story more narrative, simple, and straightforward. On the other hand, fear allows people to sympathize with the weak from a moral standpoint, allowing readers to bring the crime story into their own living environment and, simultaneously, judge the story based on their own perceived social moral standards. Finally, crime-related news stories have satisfied people’s curiosity about social disorder. Therefore, when the domestic helper topic can meet three of the six content requirements of the question frame, the news media reports will naturally use the domestic helper topic to attract attention and ratings. From this perspective, the news media does not care about FDHs, whether the helper is a victim or a perpetrator; as long as the topic can meet the content conditions of the problem frame, this topic is newsworthy.

The article’s most compelling discovery arises from Table 5. The three articles examined satisfy the content requirements of the problem frame. The noteworthy aspect is the difference in high-frequency vocabulary among the three articles. In Article 1 and Article 2, the domestic helper is the victim, while in Article 3, the domestic helper is the offender. The high-frequency word in the former is “crime,” whereas the high-frequency word in the latter is “fear.” It seems to reflect from the side that the news value of “Proximity” is affecting media coverage of FDHs (Jewkes, 2015); in other words, cultural distance determines the direction of news media reporting. In Article 3, the victim is a Hong Kong resident, and the media tries to tell the story from the point of view of the domestic helper abusing her fiercely as a child; on the one hand, because of the cultural similarity, the readers are more able to empathize with the Hong Kong resident, and on the other hand, they keep emphasizing that the victim is afraid of the domestic helper so that the “domestic helper” becomes the villain through the creation of a black-or-white role, which is a way of simplifying the story. However, when the victim becomes the domestic helper, it reduces the description of the perpetrator, instead focusing more on how the perpetrator sexually assaults the domestic helper, laying bare the explicit details of the story to the reader and then repeating the legal offense over and over again, as if the media were simply the chronicler of the case, shouldering the responsibility of publicizing the perpetrator’s crimes to the general public. These differences reflect a simple social fact. Due to cultural and geographical distance, FDHs working in Hong Kong are more often regarded as “outsiders” and “others” rather than “The Others.” The root cause of this is geographical distance, on the one hand. However, more importantly, it is the in-groups and out-groups constructed based on media news that separate Hong Kong locals from FDHs, and this actively constructed social consciousness segregation also provides the basis for xenophobia-based hate crimes.

Amidst these concerning findings, Table 6 introduces a glimmer of reassurance, indicating an annual decrease in media coverage on the “fear” topic under “domestic helper,” offering a slight respite from the prevailing negative narratives. The established problem frame practices that associate fear with FDHs may have contributed to a particular narrative fatigue or a shift in media focus, reflected in the observed decrease in coverage related to the “fear” topic.

## Conclusion

The agenda-setting of Hong Kong media is more inclined to report on the legal disputes faced by FDHs and portray them as troublemakers. At the same time, under the topic of FDHs, the agenda-setting and problem frame of Hong Kong media is more inclined to associate “fear” with FDHs rather than “rest assured,” which reflects that the Hong Kong mass media are making use of the frames of fear and problems to reshape the social image of a domestic helper. Not only that, media outlets positioned as advocates for Hong Kong locals tend to view FDHs through a lens that primarily focuses on their legal rights, inadvertently fostering a perception of these workers as outsiders; this one-dimensional portrayal lacks empathy for their broader experiences, contributing to the formation of a social consciousness that paves the way for xenophobic sentiments and potential hate crimes. Despite these prevailing narratives, an exciting shift is noted in the evolving media landscape. Over the years, there has been a noticeable decline in media coverage regarding “fear” in the context of ‘domestic helper.

In conclusion, the media’s agenda-setting and problem frame practices are pivotal in shaping public perceptions of FDHs. While the tendency to frame these workers as troublemakers and outsiders has been prevalent, the observed decline in “fear” coverage signals a potential turning point. This nuanced evolution warrants further exploration to unravel the intricate interplay between media narratives, societal attitudes, and the lived experiences of FDHs in Hong Kong.

## Data availability statement

The original contributions presented in the study are included in the article/supplementary material, further inquiries can be directed to the corresponding author.

## Author contributions

ZC: Conceptualization, Data curation, Formal analysis, Funding acquisition, Investigation, Methodology, Project administration, Resources, Software, Supervision, Validation, Visualization, Writing – original draft, Writing – review & editing.
